# Blood pressure regulation through circadian variation: PRDM16 as a target in vascular smooth muscle cells

**DOI:** 10.1172/JCI188784

**Published:** 2025-02-03

**Authors:** M. Adriana Cuibus, Omar Abdel-Wahab

**Affiliations:** Molecular Pharmacology Program, Sloan Kettering Cancer Center, New York, New York, USA.

## Abstract

The precise mechanisms of blood pressure (BP) regulation are not fully elucidated, and understanding BP regulation is crucial for managing hypertension and improving outcomes for cardiovascular disease. In this issue of the *JCI*, Wang et al. identified the transcription factor PR domain–containing protein 16 (PRDM16) as a regulator of both vascular smooth muscle cell contraction and the circadian response to BP control. PRDM16 directly transcriptionally controlled the expression of the adrenergic receptor α 1d and several clock genes crucial for BP circadian regulation. These findings identify a mechanism of how molecular pathways govern circadian BP variation, highlighting PRDM16 as a potential target for hypertension.

## Risks of dysfunctional blood pressure circadian regulation variation

High blood pressure (BP) or hypertension, is the leading cause of cardiovascular diseases (CVDs), including heart failure, coronary artery disease, atrial fibrillation, and many others (1, 2). BP follows a natural 24-hour circadian variation pattern with morning surges and nocturnal dips. Understanding the pathogenesis of abnormalities in circadian rhythm variation of BP is crucial for identifying therapeutic targets for CVD.

BP is regulated by vascular smooth muscle cell (VSMC) contraction via adrenergic receptor signaling (3). Activation of these GPCRs through various neurotransmitters and vasoactive factors leads to downstream phosphorylation of myosin light chain (MLC) proteins (4–6). Another critical aspect of BP regulation is control of BP circadian rhythm via the internal timing system of the central clock in the hypothalamic suprachiasmatic nucleus and the peripheral clock, involving several clock genes such as *Bmal1*, *Clock*, *Per*, and *Cry* (7). Links between molecular mechanisms of VSMC contraction and the internal timing system of the central clock and peripheral clock are not well understood.

In this issue of the *JCI*, Wang and colleagues hypothesized that the transcriptional regulator positive regulatory domain–containing protein 16 (PRDM16) might connect VSMC contraction and circadian rhythm regulation (8). This zinc finger transcription factor is highly expressed in VSMCs and plays a crucial role in proper arterial vascular development (9, 10). Single-cell RNA-Seq revealed that *Prdm16/PRDM16* has its most prominent expression in the aorta and artery in mice and humans (8). Further RNA-Seq analysis revealed dysregulated expression of clock genes in the aorta of VSMC-specific *Prdm16*-KO mice (*PRDM16^SMKO^*) (11), suggesting a connection between PRDM16 expression in VSMCs and BP circadian variation. Ultimately, the investigators discovered PRDM16 as a key regulator of BP circadian variation in VSMCs (8).

## *Prdm16* KO affects the physiological response and contractile response

**Figure 1:**
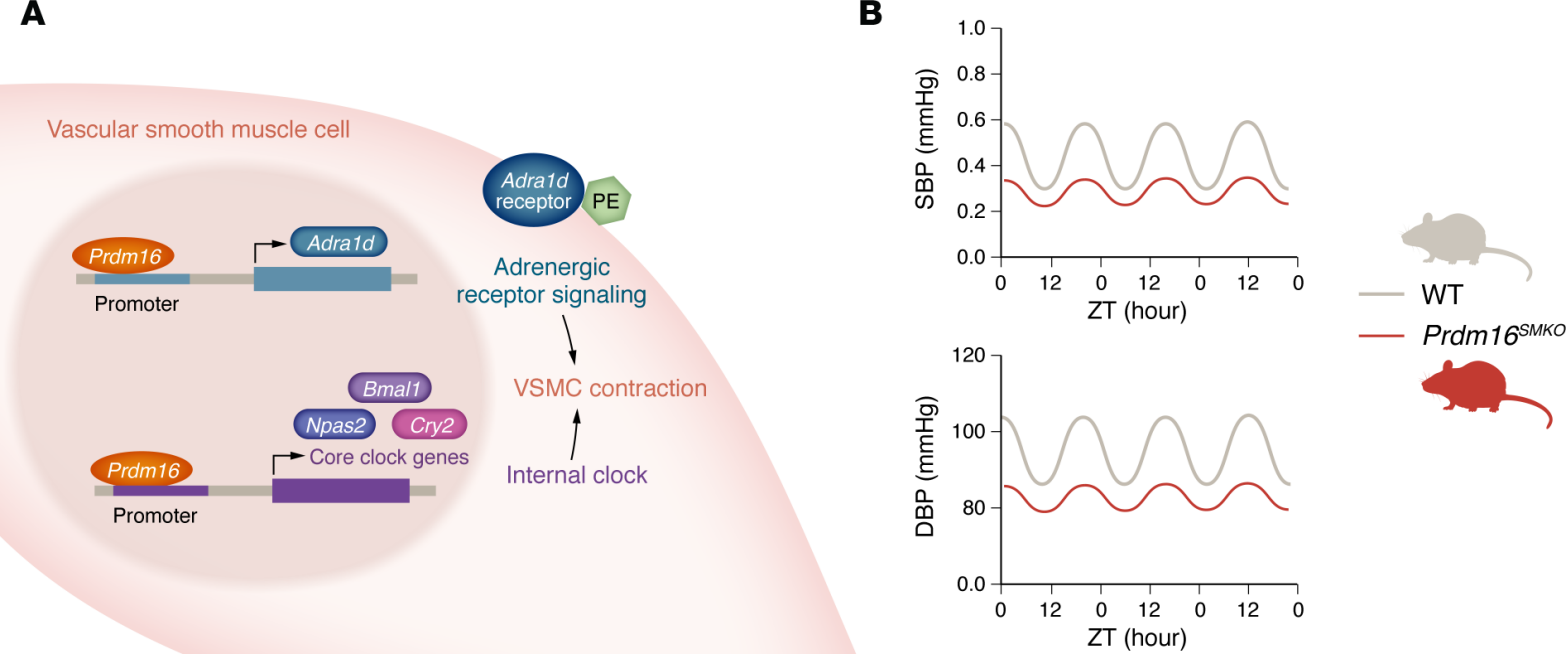
PRDM16 is a transcriptional regulator of BP circadian variation. (**A**) PRDM16 directly transcriptionally regulates expression of the ADRA1D and core clock genes *Bmal1*, *Cry2*, and *Npas2*. (**B**) Loss of PRDM16 in VASMCs leads to a nondipping, dysregulated circadian response in systolic BP (SBP) and diastolic BP (DBP). ZT, Zeitgeber time.

Wang and co-authors explored the role of PRDM16 in VSMC contraction by using *Prdm16^SMKO^* mice. Through radiotelemetry studies, *Prdm16* KO blunted systolic and diastolic BP elevation during the active phase, resulting in nondipping BP during the inactive phase and overall dysregulated BP circadian variation (Figure 1). Interestingly, heart function and a variety of aspects of metabolism were not disrupted by *Prdm16^SMKO^*. To assess how *Prdm16* KO affected the contractile response in mesenteric arteries, the authors tested various vasoconstrictors with wire myography to determine the contraction force. Among phenylephrine (PE), 55-hydroxytryptamine, prostaglandin F2α, and U46619, as well as vasodilators such as acetylcholine and sodium nitroprusside, *Prdm16^SMKO^* mice had a decreased contractile response to PE compared with control mice (8).

To elucidate the mechanism by which *Prdm16* KO drives the impaired contractile response in following treatment with PE, Wang and colleagues measured the expression of PE-related receptors. Interestingly, there was a drastic reduction in expression of the adrenergic receptor α 1d (ADRα1d) as well as of phosphorylation of its downstream effector MLC in the aorta and VSMCs of *PRDM16^SMKO^* mice. ChIP-Seq revealed that PRDM16 bound the *Adra1d* promoter, and PRDM16 was functionally confirmed to regulate *Adra1d* expression through a luciferase promoter assay (Figure 1A). The decrease in ADRα1d expression and impaired contraction response to PE in *Prdm16^SMKO^* mice reveals how PRDM16 transcriptionally regulates *Adra1d* and adrenergic receptor signaling in VSMCs.

The authors next validated the functional role of PRDM16 in the contractile phenotype through a series of 3D collagen-based cell contraction assays in rat thoracic aortas and mesenteric arteries. PRDM16 loss impeded contraction in both the thoracic aorta and mesenteric arteries and reduced expression of the well-recognized VSMC markers myosin heavy chain 11 (MYH11), smooth muscle cell α actin (αSMA), calponin 1, and smooth muscle protein 22α (SM22α) in these systems, further supporting a vital role for PRDM16 in VSMC function (8).

## PRDM16 and clock genes regulate BP circadian variation

The disrupted circadian pattern of systolic and diastolic BP in *Prdm16^SMKO^* mice suggests that PRDM16 may be linked to circadian rhythm regulation (Figure 1B). To further support this possibility, the authors found higher expression levels of *Prdm16* mRNA in the aorta during the resting phase and lower expression in the active phase. When examining the expression of clock genes over a 24-hour period in *Prdm16^SKMO^* mice, lowered *Npas2* expression notably stood out at all time points, whereas *Cry2* expression was higher at all time points in the aorta compared with the control. To test whether PRDM16 also regulates circadian gene expression, the authors performed *Prdm16* ChIP-Seq analysis, which revealed PRDM16 binding to promoters of the notable clock genes *Bmal1*, *Npas2*, *Cry1*, *Cry2*, and *Per2*. Identification of genes with altered 24-hour expression oscillation upon *Prdm16* KO that intersected with those whose promoters were bound by PRDM16 revealed *Cry2* and *Npas2* as the key target clock genes that PRDM16 regulates (8).

Finally, the investigators explored how PRDM16, ADRα1d, and circadian clock proteins regulate BP circadian variation by systematically silencing each factor and evaluating gene expression of the other members. Knockdown of one or both of the proteins encoded by the clock genes NPAS2 and BMAL1 did not affect PRDM16 or ADRα1d expression. In contrast, PRDM16 and ADRα1d each regulated the expression of NPAS2. Moreover, combined PRDM16 and ADRα1d expression synergistically reduced NPAS2 expression, suggesting that PRDM16 and ADRα1d regulated clock gene expression through a distinct pathway.

Together, Wang et al. (8) reveal a role for PRDM16 in regulating BP circadian variation by identifying a crosstalk between VSMC contraction and the internal timing system. While these findings expand the critical role of PRDM16 in the cardiovascular system and offer insight into molecular pathways that regulate BP, they raise questions about the broader implications and potential applications of this research.

## Unanswered questions

Wang et al. (8) discovered a role for PRDM16 in controlling BP and linked the two-fold regulation of VSMC contraction through the adrenergic-signaling pathway to the internal clock system (8). Although they provide evidence of how PRDM16 in VSMCs regulates the expression of *Adra1d* and core clock genes in VSMCs, some questions arise. First, the investigators uncovered an unexpected connection between ADR1AD and the regulation of clock gene expression in a PRDM16-independent manner. However, the precise connection between this GPCR and clock gene expression regulation is currently unknown. Additionally, the upstream regulators of PRDM16 circadian expression are yet to be identified. A future study could focus on establishing why no differences were observed in *Prdm16* mRNA expression upon knockdown of *Bmal1* or *Npas2* in cultured VSMCs, as previous studies have shown that *Bmal1*-KO mice had varied *Prdm16* expression across tissue types (12, 13, 14). Understanding the upstream regulators of *Prdm16* circadian expression may result in the means for pharmacological targeting of PRDM16.

## Future directions

The discovery of a role for PRDM16 in the cardiovascular system opens many avenues for future research. A leading question is how PRDM16 modulation could be used as a therapeutic intervention to restore normal BP circadian rhythms. Single nucleotide polymorphisms of PRDM16 are associated with various human diseases, leading to increased risk of migraines, obesity, and metabolic syndromes, as well as effects on heart function (15). Future studies to examine clinically relevant PRDM16 single nucleotide polymorphisms and how they might be linked to the pathogenesis driving hypertension could therefore be very enlightening. 

Additionally, in the current age of many external factors that can disrupt the 24-hour circadian rhythm, it has become increasingly essential to understand the effects of dysregulated clock genes. The mechanism for how PRDM16 regulates *Adra1d* and clock genes, while *Adra1d* independently regulates *Npas2* expression, is valuable, since it has been previously shown that dysregulated clock genes can affect cardiac metabolism in *Bmal1*-KO mice (16). This study by Wang et al. (8) also puts PRDM16 on the radar for integration into future circadian clock disruption studies.

## Version 1 02/03/2025

Electronic publication
